# Influence of Diagnostic Delay on Survival Rates for Patients with Colorectal Cancer

**DOI:** 10.3390/ijerph19063626

**Published:** 2022-03-18

**Authors:** María Padilla-Ruiz, María Morales-Suárez-Varela, Francisco Rivas-Ruiz, Julia Alcaide, Esperanza Varela-Moreno, Irene Zarcos-Pedrinaci, Teresa Téllez, Nerea Fernández-de Larrea-Baz, Marisa Baré, Amaia Bilbao, Cristina Sarasqueta, Urko Aguirre-Larracoechea, José María Quintana, Maximino Redondo, on behalf of CARESS-CCR Study Group

**Affiliations:** 1 Research Unit, Agencia Sanitaria Costa del Sol, 29603 Marbella, Spain; prmariac@gmail.com (M.P.-R.); frivasr@hcs.es (F.R.-R.); ps_evm@hotmail.com (E.V.-M.); mredondo@hcs.es (M.R.); 2 Red de Investigación en Servicios de Salud en Enfermedades Crónicas-REDISSEC, 29603 Marbella, Spain; drayulia@hotmail.com (J.A.); irenezarcos@hotmail.com (I.Z.-P.); teresatellez@yahoo.com (T.T.); nerea.fernandez@salud.madrid.org (N.F.-d.L.-B.); mbare@tauli.cat (M.B.); amaia.bilbaogonzalez@osakidetza.eus (A.B.); cristina.sarasquetaeizaguirre@osakidetza.eus (C.S.); urko.aguirrelarracoechea@osakidetza.eus (U.A.-L.); josemaria.quintanalopez@osakidetza.eus (J.M.Q.); 3 Instituto de Investigación Biomédica de Málaga (IBIMA), 29010 Málaga, Spain; 4 Unit of Public Health and Environmental Care, Department of Preventive Medicine, University of Valencia, 46100 Burjassot, Spain; 5 CIBER of Epidemiology and Public Health (CIBERESP), Institute of Health Carlos III, 28029 Madrid, Spain; 6 Servicio de Oncología Médica, Agencia Sanitaria Costa del Sol, 29603 Marbella, Spain; 7 Unidad de Gestión Clínica Intercentros de Oncología Médica, Hospitales Universitarios Regional y Virgen de la Victoria, 29010 Málaga, Spain; 8 Departamento de Especialidades Quirúrgicas, Bioquímica e Inmunología, Universidad de Málaga, 29016 Málaga, Spain; 9 Area of Environmental Epidemiology and Cancer, National Centre for Epidemiology, Instituto de Salud Carlos III (ISCIII), 28029 Madrid, Spain; 10 Clinical Epidemiology and Cancer Screening, Parc Taulí University Hospital, Parc del Taulí, 1, 08208 Sabadell, Spain; 11 Osakidetza Basque Health Service, Research Unit, Basurto Universitary Hospital, Montevideo Etorb., 18, 48013 Bilbao, Spain; 12 Kronikgune Institute for Health Services Research, 48902 Barakaldo, Spain; 13 Biodonostia Health Research Institute, Donostia Universitary Hospital, 20014 San Sebastian, Spain; 14 Research Unit, Hospital Galdakao-Usansolo, 48960 Galdakao, Spain

**Keywords:** colorectal neoplasms, delayed diagnosis, prospective cohort study, mortality, survival

## Abstract

Colorectal cancer affects men and women alike. Sometimes, due to clinical-pathological factors, the absence of symptoms or the failure to conduct screening tests, its diagnosis may be delayed. However, it has not been conclusively shown that such a delay, especially when attributable to the health system, affects survival. The aim of the present study is to evaluate the overall survival rate of patients with a delayed diagnosis of colorectal cancer. This observational, prospective, multicenter study was conducted at 22 public hospitals located in nine Spanish provinces. For this analysis, 1688 patients with complete information in essential variables were included. The association between diagnostic delay and overall survival at five years, stratified according to tumor location, was estimated by the Kaplan–Meier method. Hazard ratios for this association were estimated using multivariable Cox regression models. The diagnostic delay ≥ 30 days was presented in 944 patients. The presence of a diagnostic delay of more than 30 days was not associated with a worse prognosis, contrary to a delay of less than 30 days (HR: 0.76, 0.64–0.90). In the multivariate analysis, a short delay maintained its predictive value (HR: 0.80, 0.66–0.98) regardless of age, BMI, Charlson index or TNM stage. A diagnostic delay of less than 30 days is an independent factor for short survival in patients with CRC. This association may arise because the clinical management of tumors with severe clinical characteristics and with a poorer prognosis are generally conducted more quickly.

## 1. Introduction

Colorectal cancer (CRC) is one of the three tumors with the highest incidence worldwide, affecting men and women alike. In Europe in 2020, the estimated cumulative incidence of CRC was 519,820 cases (male and female). This cancer was the most frequently diagnosed in Spain, and 43,581 new cases were forecast to appear in 2021. Although overall age-standardized mortality rates from cancer are declining, the global number of CRC deaths is increasing and the estimated number of deaths for 2020 was 935,173, representing 9.4% of all cancer-related deaths and second only to lung cancer. In Spain, 16,470 deaths from CRC were estimated for the same year [1,2].

In recent years, CRC screening programs have become increasingly common in Spain, making it possible to treat the disease at an earlier stage and thus favoring patients’ prognosis and survival. However, although the general coverage of these programs is expanding, in response to the 2003 recommendations on cancer screening made by the European Commission and within the National Strategy against Cancer, they have yet to reach 100% of the population at risk [3]. This shortcoming is due, at least in part, to the fact that implementation of screening programs started in different years depending on the region (Autonomous Community). In consequence, a relatively high percentage of patients are still only diagnosed when the symptoms become evident, at more advanced stages of the disease [4,5,6,7].

The initial delay, from the appearance of symptoms until the patient consults with the referring physician, is termed the patient-dependent delay. The subsequent passage of time, until diagnostic tests are performed and the pathological results obtained, is termed the diagnostic delay [8]. Previous studies of cohorts of patients have analyzed the factors associated with both types of delay [9,10]. Some authors have reported that late diagnosis does not directly affect mortality from CRC, which is related to other clinical and sociodemographic factors [11,12]. According to a recent article concerning a retrospective cohort study conducted at a single hospital, short diagnostic delays are significantly associated with a poorer prognosis, an effect that is called the “waiting time paradox” [13]. In view of these considerations, our study aim is to evaluate the overall survival rate of CRC patients in relation to diagnostic delay, based on the prospective analysis of a large cohort (CARESS/CCR Study) [14]. 

## 2. Methods

### 2.1. Study Design

**Figure 1:**
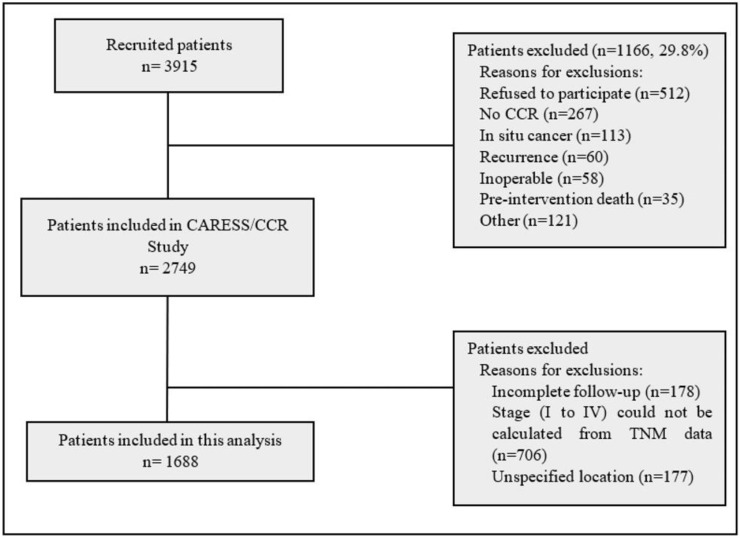
Flowchart summarizing the inclusion and exclusion criteria applied.

This prospective observational cohort study was carried out at 22 hospitals (all belonging to the Spanish National Health System) located in nine provinces of Spain. The study included 2749 patients who were diagnosed for the first time as CRC and underwent surgery between June 2010 and December 2012, and who were then followed up for five years. Neither recurrences nor metastases have been included (Figure 1). The patients were recruited prospectively, and relevant sociodemographic and clinical information was obtained from the hospital databases and by self-reported questionnaires [14,15]. 

### 2.2. Inclusion and Exclusion Criteria

Figure 1 shows the flowchart of participants in the study and reasons for exclusion. For the present analysis, we only included patients with complete information on five-year survival, TNM stage and tumor location (Figure 1). 

### 2.3. Study Variables

Data were collected for the patient’s sociodemographic variables (age, sex, education, home situation), personal history (BMI, Charlson comorbidity index, smoking habit, family history of CRC) and tumor-related variables (location, stage, degree of differentiation, histological diagnosis and screening diagnosis). According to the CRC protocols applied in most Spanish hospitals, a diagnostic delay of less than 30 days is considered an indicator of good quality [16]. Data on life status at five years after diagnosis were obtained from hospital databases, patient/family questionnaires and the National Death Index. Survival time was calculated as the difference between the date of death for any cause and the date of diagnosis.

The data for first medical consultation were derived from the date of the first visit to the hospital or of the date of screening. The date of diagnosis was taken as the date of the pathology report. When the diagnosis could not be performed by histology the dates of CT or MRI were used.

### 2.4. Ethical Considerations

The project was approved by the corresponding research ethics committees. The study data were recorded anonymously, in strict accordance with applicable data protection laws and regulations. All participants signed an informed consent. This project was approved by the following Ethics Committees in Spain (reference number of approval, when provided, in brackets): the Ethics Committees of the Hospitals of Txagorritxu (2009–20), Galdakao, Donostia (5/09), Basurto and Marbella (10/09), and the Ethics Committee of the Basque Country (PI2014084).

### 2.5. Statistical Plan

The descriptive analysis was performed using measures of central tendency and dispersion for the quantitative variables and of frequency distribution for the qualitative ones. A bivariate analysis was performed to assess differences in sociodemographic and clinical variables depending on the presence of a diagnostic delay. Student’s t test was used for the quantitative variables, and the chi-squared test was used for the qualitative ones. Subsequently, survival analysis was performed using the Kaplan–Meier method, taking as a segmentation variable the presence of diagnostic delay (including probable DX), stratified according to tumor location. Differences were evaluated using the Mantel–Cox log-rank test. Finally, crude and multivariate Cox regression models were constructed to select the most parsimonious model. Hazard ratios were described, with the respective 95% confidence intervals. In the multivariate Cox model, the significant variables were initially included in the crude analysis. For all analyses, the level of statistical significance assumed was *p* < 0.05. The statistical program used was SPSS v.15.(IBM Corp, Armonk, NY, USA) 

## 3. Results

The study sample consisted of 1688 CRC patients who had information on their diagnostic delay and tumor location, and who were subsequently followed up for five years. The patients’ mean age at diagnosis was 68 years, and 63.6% were male. According to the Charlson index, these patients presented an average of 2.8 (SD 1.2) comorbidities. Most of them had tumors in stage II or III (35.2% and 31.5%, respectively) (Table 1).

Within this sample of patients, the median diagnostic delay was 36.5 days (interquartile range: 73), and 55.9% (*n* = 944) experienced a diagnostic delay ≥30 days (95% confidence interval: 53.5–58.3). BMI was positively associated with the delay (*p* = 0.028), while the Charlson index was inversely associated with it (*p* = 0.035). The patients who were diagnosed as the result of a screening were more likely to experience a delay ≥ 30 days (*p* < 0.001) (Table 1).

**Figure 2:**
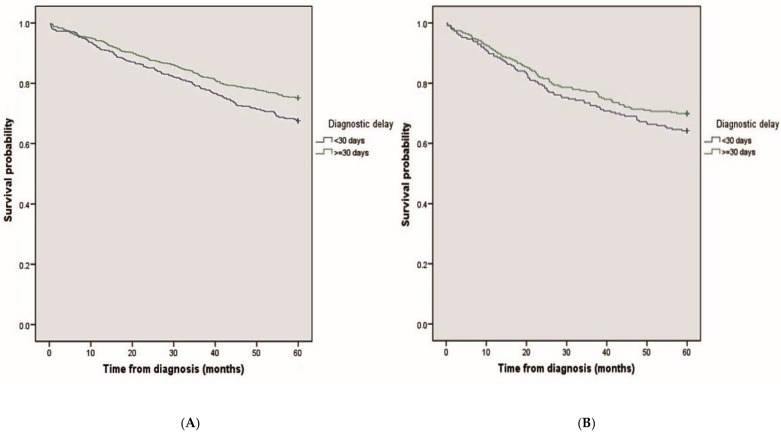
Survival adjusted by tumor location. (**A**) Left colon/rectum; (**B**) right colon.

The above differences in survival rates according to the presence or otherwise of diagnostic delay persisted when the patients were stratified by tumor location (Figure 2A, left colon/rectum, B, right colon) (see also Table 2). 

With the exception of smoking habit, crude Cox regression analysis revealed a significant association between overall survival and all of the sociodemographic and clinical characteristics included in the analysis, including the presence of diagnostic delay (HR: 0.76; 95% CI: 0.64–0.90).

In the subsequent multivariate model, the presence of diagnostic delay persisted as a factor associated with a better prognosis (HR: 0.80; 95% CI: 0.66–0.98), regardless of age (HR: 1.04), BMI (HR: 1.02), Charlson index (HR: 1.30) and TNM stage: a HR of 9.38 was found for stage IV CRC (Table 3).

## 4. Discussion

The finding provides further evidence of a lack of a consistent relationship between diagnostic delay and survival. In a prospective cohort of patients with CRC with a five-year follow-up, our analysis detected a paradoxical relationship between diagnostic delay and survival. Thus, patients who experienced a longer diagnostic delay (more than 30 days) had a better medium-term prognosis, regardless of the sociodemographic, clinical and biological characteristics of the tumor.

In previous research in this field, diverse criteria have been applied regarding diagnostic delay; thus, in general, the diagnostic delay intervals considered in our series were shorter than those reported elsewhere [17]. Nevertheless, our results corroborate those of other published studies in that a longer delay is not associated with a worse prognosis in terms of overall survival [11,18,19,20]. On the other hand, some controversy remains, since another study has reported the existence of a worse prognosis associated with diagnostic delay [21]. On balance, however, our main result confirms the paradoxical relationship highlighted by Pita-Fernández et al., according to which diagnostic delay is a protective factor, significantly affecting the survival of patients with CRC. This paradoxical effect is maintained independently of the tumor location and stage [13].

In a prior study, focusing on patients with breast cancer, we observed the same relationship between diagnostic delay and survival. Thus, shorter delays in diagnosis were significantly associated with advanced stages of the disease and low survival rates. This paradoxical relationship may be due to the fact that the sickest patients usually receive immediate medical attention [22]. Thus, in our study, symptomatic patients, contrary to those detected by screening, had a very significantly shorter delay.

Furthermore, we suggest that the interpretation of the present results may also benefit from following conceptual considerations. Colon cancer prognosis could be largely influenced by factors active in the presymptomatic phases of the disease, and the symptomatic phase could represent a much smaller fraction of the natural history of malignancy. A limitation of this study has been the fact of not being able to calculate the stage (I to IV) in a significant percentage due to the lack of some data in the TNM (Figure 1). Nevertheless, the study sample is large enough to answer the questions raised (*n* = 1688). Moreover, in our study the tumor stage was the best prognostic predictor, which suggests that the data collection process was of adequate quality. Another aspect to take into account in relation to the results of this study is the possibility that the concept of early detection should or could be measured in terms of the stage of the tumor rather than the duration of symptoms.

## 5. Conclusions

In conclusion, diagnostic delay was associated with better overall survival in patients with CRC. This is probably because tumors with a poorer prognosis are clinically managed in a preferential way; by contrast, tumors believed to be less aggressive, given their clinical characteristics, are likely to present a longer delay.

## Figures and Tables

**Table 1:** Sociodemographic and clinical characteristics for the total sample and segmented by diagnostic delay.

	Total		Diagnostic delay				*p*
			<30 days		≥30 days		
	n: 1688	% *	n: 744	% **	n: 944	% **	
**Sex**							
Male	1073	63.6	469	43.7	604	56.3	0.726
Female	615	36.4	275	44.7	340	55.3	
**Age ^1^**							
*Mean-SD*	68.0	10.8	68.4	10.7	67.7	10.9	0.164
**Education ^2^**							
Primary or less	1082	76.3	466	43.1	616	56.9	0.738
Secondary–university	337	23.7	141	41.8	196	58.2	
**Habitation status ^3^**							
Living alone	198	14.0	86	43.4	112	56.6	
With family	1180	83.5	504	42.7	676	57.3	0.194
Care home/other situations	36	2.5	10	27.8	26	72.2	
**BMI ^4^**							
*Mean-SD*	27.0	6.9	26.5	7.4	27.4	6.5	0.028
Charlson index							
*Mean-SD*	2.8	1.2	2.9	1.3	2.8	1.2	0.035
**Smoking habit ^5^**							
Never smoked	759	47.2	335	44.1	424	55.9	
Current smoker	213	13.2	91	42.7	122	57.3	0.933
Ex-smoker	637	39.6	280	44.0	357	56.0	
**Family history of CRC ^6^**							
No	857	84.4	347	40.5	510	59.5	0.722
Yes	158	15.6	61	38.6	97	61.4	
**Tumor location**							
Right colon	502	29.7	226	45.0	276	55.0	
Left colon	726	43.0	311	42.8	415	57.2	0.673
Rectum	460	27.3	207	45.0	253	55.0	
**TNM stage**							
I	374	22.2	148	39.6	226	60.4	0.295
II	595	35.2	271	45.5	324	54.5	
III	531	31.5	247	46.5	284	53.5	
IV	188	11.1	78	44.1	110	58.5	
**Degree of differentiation ^7^**							
Low	1263	85.9	560	44.3	703	55.7	0.212
High	207	14.1	102	49.3	105	50.7	
**Histologic diagnosis ^8^**							
Adenocarcinoma	1495	91.0	650	43.5	845	56.5	0.056
Mucinous adenocarcinoma or others	148	9.0	77	52.0	71	48.0	
**Screening diagnosis ^9^**							
Absent	1301	81.4	610	46.9	691	53.1	<0.001
Present	297	18.6	90	30.3	207	69.7	

* Percentage by columns; ** percentage by rows; losses: ^1^ = 1; ^2^ = 269; ^3^ = 274; ^4^ = 346; ^5^ = 79; ^6^ = 673; ^7^ = 218; ^8^ = 45; ^9^ = 90.

**Table 2:** Survival analysis according to diagnostic delay, stratified by location.

		Mean Survival (Months) 95% CI	*p*
**Overall**		49.8 (49.0–50.7)	
**diagnostic delay**			
	<30 days	48.6 (47.3–50.0)	*0.002*
	≥30 days	50.8 (49.6–51.9)	
**Location**			
Right colon	<30 days	46.8 (44.2–49.4)	*0.002*
	≥30 days	48.6 (46.4–50.9)	
Left colon + rectum	<30 days	49.4 (47.9–51.0)	
	≥30 days	51.6 (50.4–52.9)	

**Table 3:** Crude and adjusted overall survival analysis using the Cox model.

	Crude		Adjusted *	
	*p*	HR 95%CI	*p*	HR
**Diagnostic delay**				
<30 days	*0.002*	1.00	*0.034*	1.00
≥30 days		0.76 (0.64–0.90)		0.80 (0.66–0.98)
**Sex**				
Male	*0.013*	1.00		
Female		0.79 (0.65–0.95)		
**Age**				
	*<0.001*	1.04 (1.03–1.05)	*<0.001*	1.04 (1.03–1.05)
**Education**				
Primary or less	*0.004*	1.00		
Secondary–university		0.69 (0.53–0.88)		
**Habitation status**				
Living alone		1.00		
With family	0.043	0.78 (0.60–1.02)		
Care home / Other situations		1.29 (0.73–2.25)		
**BMI**				
	*0.016*	1.02 (1.00–1.04)	*<0.018*	1.02 (1.00–1.04)
**Charlson index**				
	*<0.001*	1.30 (1.23–1.37)	*<0.001*	1.30 (1.22–1.38)
**Smoking habit**				
Never smoked		1.00		
Current smoker	0.881	1.03 (0.77–1.36)		
Ex-smoker		1.05 (0.87–1.28)		
**Family history of CRC**				
No	*0.017*	1.00		
Yes		0.65 (0.46–0.93)		
**Tumor location**				
Right colon	*0.033*	1.00		
Left colon + rectum		0.82 (0.68–0.98)		
**TNM stage**				
I	*<0.001*	1.00	*<0.001*	1.00
II		1.57 (1.14–2.16)		1.47 (1.02–2.12)
III		2.86 (2.11–3.88)		3.06 (2.16–4.33)
IV		8.38 (6.08–11.5)		9.38 (6.46–13.6)
**Degree of differentiation**				
Low	*<0.001*	1.00		
High		1.63 (1.28–2.07)		
**Histologic diagnosis**				
Adenocarcinoma	*<0.001*	1.00		
Mucinous adenocarcinoma or others		1.63 (1.24–2.13)		

* Multivariate Cox model. Sample: 1342 patients.

## Data Availability

The data presented in this study are available on request from the corresponding author. The data are not publicly available due to patient confidentiality.
